# Why and When Do Good Soldiers Behave Unethically? Introducing Conservation of Resources Theory to Explain the Curvilinear Effects of Organizational Citizenship Behavior

**DOI:** 10.3389/fpsyg.2021.619657

**Published:** 2021-07-28

**Authors:** Fangfang Xu, Shiyong Xu, Jinqiang Zhu, Jinyi Zhou, Bainan Zhang, Chunmeng Yang

**Affiliations:** ^1^School of Labor and Human Resources, Renmin University of China, Beijing, China; ^2^Center for Human Resource Development and Assessment, School of Labor and Human Resources, Renmin University of China, Beijing, China; ^3^School of Management, Minzu University of China, Beijing, China; ^4^Department of Business Administration, Donlinks School of Economics and Management, University of Science and Technology Beijing, Beijing, China

**Keywords:** organizational citizenship behavior, citizenship fatigue, perceived organizational support, counterproductive work behavior, conservation of resources theory

## Abstract

Previous research about organizational citizenship behavior (OCB) and counterproductive work behavior (CWB) has produced contradictory results. Drawing from the conservation of resources (COR) theory, the present study tries to explain the contradictory findings by examining the curvilinear relationship between OCB and CWB. Using data collected at three time points from 426 employees and 110 supervisors in Chinese companies, data analysis shows that OCB has an inverted U-shaped relationship with CWB. The results also demonstrate that citizenship fatigue mediates the relationship between OCB and CWB, perceived organizational support (POS) moderates the relationship between OCB and citizenship fatigue. In addition, POS moderates the mediating effect of citizenship fatigue in the inverted U-shaped curvilinear relationship between OCB and CWB. This mediating effect is stronger under conditions of low POS than high POS. The findings present a complementary explanation of the conflicting relationships between OCB and CWB.

## Introduction

Organizational citizenship behavior (OCB) and counterproductive work behavior (CWB) have been two of the most widely researched constructs in organizational behavior over the past 30 years (Organ, 2018; Yuan et al., 2018; Bauwens et al., 2019; Sypniewska, 2020; Wang et al., 2021). OCB refers to employee behavior that goes beyond role requirements and contributes to organizational effectiveness but is often discretionary and not rewarded relative to in-role job performance, an example of OCB is helping colleagues (Organ, 1988, 1997; Smith et al., 1983). Individuals who engage in OCB may be referred to as “good soldiers” (Organ, 1988). Conversely, CWB describes acts that are harmful to the organization by directly affecting its functioning or property, or by hurting employees in a way that reduces their effectiveness, such as being rude (Fox et al., 2001; Sackett, 2002). CWB is considered as a special kind of unethical behavior. Considering the opposite definitions and significant impacts that OCB and CWB have on organizations and employees, much of the literature and managerial practices has focused on measures to simultaneously increase the level of OCB and reduce the level of CWB (e.g., Zhang et al., 2019). However, this may be difficult because the studies theorizing about the relationship between OCB and CWB have shown that their relationship is complicated and may not be a simple negative correlation. In fact, there is still heated debate on the relationship and underlying mechanism between OCB and CWB.

Specifically, some studies, especially meta-analytic research have found that OCB and CWB are negatively linearly correlated (e.g., Dalal, 2005) or have no significant relationship (e.g., Miles et al., 2002). These studies were mainly based on the opposite structures, the presence of antithetical items, and distinct patterns of relationships with antecedents that OCB and CWB exhibited (e.g., Dalal, 2005). They reported limitations about the mechanism of OCB on CWB (e.g., Fox et al., 2012). More recently, a few studies have paid attention to why engaging in OCBs sometimes motivates people to subsequently perform CWBs and have theorized on the underlying mechanisms. Spector and Fox (2010b) posited that OCB may actually lead to CWB when employees resent being compelled to perform OCBs because of incompetent coworkers, organizational constraints, or supervisor demands. They further suggest that employees who resent not having been rewarded for OCBs feel angry and are more likely to engage in CWB (Spector and Fox, 2010a). In addition, based on moral licensing theory (Miller and Effron, 2010), Klotz and Bolino (2013) suggest that individuals gain a moral license when they engage in morally praiseworthy behavior (e.g., OCB), which allows people to carry out negative acts (e.g., CWB) without publicly discrediting themselves. Some studies have provided empirical evidence for these claims (Yam et al., 2017; Ahmad et al., 2020; Griep et al., 2021; Lowery et al., 2021). For example, Yam et al. (2017) argue that when employees feel compelled to engage in OCB due to external forces, such as informally required by a supervisor, they are more likely to feel psychologically entitled to engage in CWB. Although these explanations are helpful for understanding why OCB has a positive effect on CWB under some boundary conditions, they fail to account for the negative or insignificant correlation between OCB and CWB found in past research (Miles et al., 2002).

Indeed, researchers have argued that OCB is both a resource-depleting and resource-acquiring activity that produces opposite effects on individuals’ psychological state and work behavior (e.g., Bolino and Klotz, 2015; Koopman et al., 2016; Organ, 2018). Obviously, employees can benefit from engaging in OCB because it inspires reciprocity which facilitates the acquisition of resources from colleagues and leaders (Koopman et al., 2016). On the other hand, OCB has costs and side effects (Deery et al., 2017), engaging in OCB may increase stress reactions because it involves investing cognitive, emotional, and physical resources (Bolino and Turnley, 2005; Bolino et al., 2015; De Clercq et al., 2019). These opposite influences might cancel each other out and explain the inconsistent findings (positive, negative and non-significant correlation) reported between OCB and CWB (e.g., Miles et al., 2002). Thus, more work is needed to understand how OCB relates to CWB.

**FIGURE 1:**
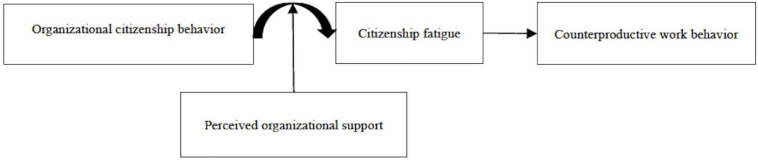
The proposed research model.

Using conservation of resources (COR) theory (Hobfoll, 1989, 2001; Hobfoll et al., 2018) as a framework, we aim to examine the curvilinear (inverted U-shaped) relationship between OCB and CWB as well as the mediating process and boundary condition associated with this relationship. First, we contend that citizenship fatigue is an important mediator in the relationship between OCB and CWB. Citizenship fatigue is a state of cognition and affect characterized by “feeling worn out, tired, or on edge” because of engaging in OCB (Bolino et al., 2015). We argue that when the levels of OCB increase from low to moderate, it may increase citizenship fatigue because stress occurs with the depletion of energies when engaging in OCB (Bolino et al., 2015; Klotz et al., 2018; Qiu et al., 2020), which drives individuals to engage in CWB. We also suggest that when the levels of OCB increase from moderate to high, it exerts the resource acquisition motivation, by engaging in OCB to benefit from resource gains, citizenship fatigue decreases, which ultimately restrains CWB (Hobfoll, 1989, 2001; Hobfoll et al., 2018). Second, according to COR theory, we further propose that the curvilinear effect of OCB on citizenship fatigue is moderated by perceived organizational support (POS)—employees’ generalized perception of the extent to which the organization values their contributions and cares about their well-being (Eisenberger et al., 1986; Eisenberger and Stinglhamber, 2011; Kurtessis et al., 2017). We suggest that the initial personal resources provided by an organization have a significant impact on the processes of resource conservation and resource acquisition (Hobfoll, 1989, 2001; Hobfoll et al., 2018). In sum, we advance the literature on the relationship between OCB and CWB by examining an integrated model that explains when (POS) and why (citizenship fatigue) good soldiers behave unethically (see Figure 1).

By testing this curvilinear, moderated mediation model of OCB and CWB, we seek to make three theoretical contributions to the literature. First, we aim to bridge the conflicting and paradoxical research findings regarding the relationship between OCB and CWB, which has been reported as various types of linear correlation (e.g., Dalal, 2005). Specifically, drawing on COR theory (Hobfoll, 1989, 2001; Hobfoll et al., 2018), we attempt to explore the curvilinear relationship between OCB and subsequent CWB. By doing so, we offer a novel perspective for future research on ex-role behavior research. We also answer the call for research exploring whether there is an optimal level of OCB and understanding how organizations can properly manage and balance both OCB and CWB in the workplace (Bolino and Klotz, 2015).

Second, we answer a recent call to examine the role of resource depletion in the relationship between OCB and CWB (Bolino et al., 2013; Bolino and Klotz, 2015; Koopman et al., 2020). In contrast to prior studies’ focus on the mediating roles of emotion (e.g., Spector and Fox, 2010a) and moral aspects (e.g., Yam et al., 2017), we identify citizenship fatigue as the resource-based mediator explaining why OCB affects CWB. The idea that OCB contains both resource-depleting and resource-acquiring aspects suggests a non-linear relationship between OCB and citizenship fatigue and subsequent CWB. We contribute to a burgeoning stream of research examining the mediating role of citizenship fatigue, which has been proven to be an important mediator in the relationship between citizenship pressure and job performance (De Clercq et al., 2019) and thriving at work (Qiu et al., 2020).

Third, by identifying the moderating role of POS, we address recent calls to clarify its influence as a protective factor against resource loss and fatigue (She et al., 2019) and in promoting an organizational environment where employees can engage in OCB in a way that maximizes its positive aspects and minimizes it negative aspects (Garcia et al., 2021). We demonstrate that POS can weaken the curvilinear relationship between OCB and citizenship fatigue. We expand POS literature by disclosing its key role in reducing stress and replenishing employee resources in the context of resource loss (Hobfoll et al., 2018).

### Theory and Hypotheses

#### Conservation of Resources Theory: Resource Conservation Tenet and Resource Acquisition Tenet

COR theory suggests that individuals have a tendency to strive to obtain, retain, foster, and protect those resources they centrally value (Hobfoll, 1989, 2001; Hobfoll et al., 2018). COR theory consists of two tenets, “resource conservation” and “resource acquisition,” which presents individuals’ contrasting consequences regarding resource losses in the workplace. On the one hand, the resource conservation tenet argues that when people lose resources at work, they are more likely to experience strain (Hobfoll, 1989, 2001; Hobfoll et al., 2018). It also posits that resource loss is disproportionately more salient than resource gain, and the impact of resource loss tends to be much greater and faster than the impact of resource gain (Hobfoll, 2001; Hobfoll et al., 2018). By contrast, the resource acquisition tenet argues that people must invest resources to protect against resource loss, to recover from loss, and to gain resources (Hobfoll, 2001; Hobfoll et al., 2018). COR theory also posits that stress emerges (1) when individuals’ resources are threatened with loss, (2) when individuals’ resources are actually lost, and (3) when individuals fail to gain sufficient resources after significant effort (Hobfoll, 1989, 2001; Hobfoll et al., 2018).

Additionally, COR theory proposes the gain paradox principle, which states that resource gain increases in salience in the context of resource loss (Hobfoll et al., 2018). Second, resource loss spirals corollary argues that resource loss is more powerful than resource gain, and because stress occurs when resources are lost, individuals and organizations have fewer resources to offset resource loss at each iteration of the stress spiral (Demerouti et al., 2004; Hobfoll et al., 2018).

#### Organizational Citizenship Behavior and Citizenship Fatigue

Based on the two tenets of COR theory, we suggest that OCB has an inverted U-shaped relationship with citizenship fatigue. According to the resource conservation tenet (Hobfoll, 1989, 2001; Hobfoll et al., 2018), when people lose resources at work, they are more likely to experience job burnout and negative physical or psychological impacts (Halbesleben et al., 2014; Baranik et al., 2019; Maffoni et al., 2020; Merino et al., 2021). Therefore, we argue that when the level of OCB increases from low to moderate, individuals are confronted with the increasing loss of critical resources (e.g., time, cognitive resources, and emotional resources) (Bergeron, 2007), they lack the energy to maintain their normal functioning at OCB, eventually leading to the increase in citizenship fatigue (Bolino et al., 2015; Qiu et al., 2020). In addition, research has demonstrated that it is difficult for employees to gain new resources when they perform low levels of OCB, because they have not established reciprocity yet and can hardly gain the social resources (e.g., social capital) through the low levels of social interactions with colleagues (Rapp et al., 2013), and this will result in exhaustion and burnout (Vîrgă et al., 2020).

By contrast, according to the resource acquisition tenet, gain paradox principle and resource loss spiral corollary (Hobfoll, 2001; Demerouti et al., 2004; Hobfoll et al., 2018), we propose that when the level of OCB increases from moderate to high, employees will acquire new resources to stop further resource loss spiral. We argue that employees are more eager to invest resources in the context of resource loss (Hobfoll et al., 2018), because if they are not trying to acquire new resources, they may further enter an escalating spiral of losses, which leads to further health impairment (Hobfoll et al., 2018). Specifically, studies have noted that OCB is a positive interpersonal activity that likely builds psychological resources by fulfilling basic human needs such as autonomy and relatedness (Bono et al., 2013) and enhancing self-evaluation (Weinstein and Ryan, 2010). Some scholars found higher levels of OCB were associated with higher levels of work meaningfulness and personal resources in the form of vigor (Lam et al., 2016), and low negative states such as stress or strains (Koopman et al., 2016). Halbesleben and Bowler (2007) found that engaging in more OCBs targeted at their coworkers could slow additional resource loss and buffer employees’ emotional exhaustion, because they can develop relationships with others to gain back resources.

In sum, consistent with the curvilinear research investigating the stressor-behavior relationship (Qin et al., 2014; Zhou et al., 2019), we suppose that an OCB inflection point exists that transforms resource conservation tenet to resource acquisition tenet. When OCB increases but is lower than the inflection point, resource conservation tenet drives employees to conserve their resources and experience the citizenship fatigue. Thus, the increase of OCB will lead to an increase in citizenship fatigue. When OCB reaches the inflection point, the requirements for acquiring additional resources to offset resource loss begin to outweigh the fear of resource loss (Qin et al., 2014). When OCB increases past this inflection point, employees cannot withstand any further resource losses, they acquire new resources to stop resource loss spirals based on the resource acquisition tenet. Hence, the increase in OCB results in a decrease in citizenship fatigue. Therefore, we propose the following hypothesis:

*Hypothesis 1. There is an inverted U-shaped relationship between OCB and citizenship fatigue.*

#### The Moderating Role of Perceived Organizational Support

The core element of POS is “the degree to which employees feel supported by their organization” (Eisenberger et al., 1986). POS is a valuable type of resource, which includes material resources (e.g., wages) and emotional resources (e.g., respect) provided by the organization (Kurtessis et al., 2017; She et al., 2019). POS influences how individuals react to resource loss and resource investment (Bolino et al., 2015; Garcia et al., 2021). Prior empirical studies have demonstrated that POS can help individuals reduce resource loss and deal with stress, given that it can replenish their resources (e.g., vigor, self-efficacy; Ott et al., 2019) and reduce burnout (Leupold et al., 2020). We suggest POS has an impact on the curvilinear relationship between OCB and citizenship fatigue.

According to COR theory, stress is unlikely to occur when individuals have enough resources to cope with the stress and challenges (Hobfoll, 1989). Therefore, we expect our proposed inverted U-shaped relationship between OCB and citizenship fatigue may not hold for all people. For employees with high POS, we suggest that OCB is not related to citizenship fatigue. As COR theory posits, individuals with greater resources are less susceptible to resource loss and more capable of acquiring new resources given their tendency toward resource accumulation, namely a tendency toward resource caravans (Hobfoll et al., 2018). Resource caravans allow individuals to maintain high levels of well-being, which can foster the acquisition of additional job resources, leading to a so-called gain spiral (Hobfoll et al., 2018). Environmental conditions that support, foster, enrich, and protect the employee’s resources (e.g., POS) can also stimulate the gain spiral (Hobfoll et al., 2018). Therefore, we expect that employees with high POS have a resource advantage over those with low POS when engaging in OCB. Employees with high POS perceive that they are rich in personal resources and do not feel exhausted and tired. People with a pool of resources to draw from also have a greater opportunity to invest resources (Halbesleben et al., 2014). Thus, for those people, engaging in OCB will not produce citizenship fatigue (Bolino et al., 2015).

Conversely, for employees with low POS, we propose that OCB has an inverted U-shaped relationship with citizenship fatigue. According to COR theory, individuals who lack resources are more vulnerable to resource loss and less capable of resource gain (Hobfoll et al., 2018). We suggest that individuals with low POS will diminish their perceptions of resource availability, thereby strengthening their decision to conserve and invest resources (Halbesleben et al., 2014; Halbesleben and Wheeler, 2015). As such, when engaging in OCB, employees with low POS will have difficulties in halting resource loss and replenishing resources (Halbesleben et al., 2014). Accordingly, they are more likely to conserve their resources and thus feel more exhausted than those with high POS. At the same time, they are more motivated to save their resources and obtain resources in the context of resource loss (Hobfoll et al., 2018). According to the resource acquisition tenet, when OCB reaches high levels, employees with low POS gain new resources (e.g., work meaningfulness and vigor) and their citizenship fatigue will decline (Hobfoll et al., 2018). Thus, we posit the following:

*Hypothesis 2. POS moderates the relationship between OCB and citizenship fatigue, such that the relationship is insignificant when POS is high but inverted U-shaped when POS is low.*

#### Citizenship Fatigue and Counterproductive Work Behavior

In our study, because citizenship fatigue is primarily characterized by the affective state of being tired, frustrated, and on edge (Bolino et al., 2015), it represents the state of resource loss. According to the resource desperation principle of COR theory, when people are overstretched or exhausted, they enter a defensive mode to preserve the self, in which they are often aggressive and may become irrational (Hobfoll et al., 2018). These individuals’ aggressive or seemingly irrational responses may work at that time because they can potentially alter the array of stressors or allow for a new coping strategy to emerge (Hobfoll and Shirom, 2001; Halbesleben et al., 2014; Hobfoll et al., 2018).

The fact that employees become more defensive in their resource investment strategies when they lose resources has been widely supported (Halbesleben and Wheeler, 2015; Maffoni et al., 2020; Sommovigo et al., 2020). In addition, an increasing body of research has demonstrated that resource depleted employees may engage in unethically behaviors, such as workplace deviance (Christian and Ellis, 2011), cheating (Welsh and Ordóñez, 2014), need-related unethical behavior (Yam et al., 2014), customer sabotage (Shao and Skarlicki, 2014) and incivility (Sommovigo et al., 2020). Therefore, we suggest that citizenship fatigue will motivate individuals to engage in CWB, and propose the following hypothesis:

*Hypothesis 3. There is a positive linear relationship between citizenship fatigue and CWB.*

#### The Mediating Role of Citizenship Fatigue

Recently, researchers have paid attention to investigating why OCB leads to CWB. Some research focused the role of negative affect (e.g., anger) in the relationship between OCB and CWB, arguing that employees who resent not having been rewarded for OCBs feel angry and are more likely to engage in CWB (e.g., Spector and Fox, 2010a). Some studies emphasized the positive relationship between OCB and CWB from the perspective of moral licensing, arguing that individuals gain a moral license when they engage in OCB, which allows people to carry out CWB without publicly discrediting themselves (e.g., Yam et al., 2017). However, these studies did not reconcile the inconsistent findings (e.g., the negative or insignificant correlation between OCB and CWB) found in prior studies (e.g., Miles et al., 2002; Dalal, 2005).

In addition, some studies have suggested that it is necessary to explore the impact of OCB on CWB from the perspective of resource depletion. For instance, Bolino and Klotz (2015) suggested that engaging in OCB often leaves employees feeling stressed and emotionally exhausted. When employees’ resources are depleted, they are especially likely to engage in CWB, such as withdrawal behaviors (Maffoni et al., 2020). Some studies have also found that when people engaging in helping behavior, employees must allocate resources to take on additional responsibilities, by switching focus from in-role behaviors to an off-task demand, which leads to resource depletion and subsequent incivility (Xiao et al., 2021) and deviance (Koopman et al., 2020). Therefore, although empirical research has yet to link OCB to CWB through the mechanism of resource, there is compelling evidence that this is likely one of the primary paths through which good soldiers may behave unethically (Bolino and Klotz, 2015). We suggest that OCB has an influence on CWB through citizenship fatigue.

Consistent with the above discussion, we further suggest that the inverted U-shaped relationship between OCB and citizenship fatigue will extend to the indirect relationship between OCB and CWB through citizenship fatigue. As stated earlier, according to the resource conservation tenet (Hobfoll, 1989, 2001; Hobfoll et al., 2018), when the level of OCB changes from low to moderate, employees’ level of citizenship fatigue increases, which motivates employees to engage in CWB. However, when the level of OCB changes from moderate to high, the resource acquisition tenet begins to play its role (Hobfoll, 1989, 2001; Hobfoll et al., 2018). By gaining new resources, the level of citizenship fatigue decreases and thus the level of CWB declines. Therefore, we propose the following hypothesis:

*Hypothesis 4. Citizenship fatigue plays a mediation role in the inverted U-shaped relationship between OCB and CWB.*

#### The Curvilinear Moderated Mediation Model

Based on the above arguments, we further suggest that POS will moderate the mediating effect of citizenship fatigue between OCB and CWB. According to the COR theory, employees with high POS will not feel pressure because of sufficient personal resources (Hobfoll et al., 2018). Thus, as discussed above, their increase in OCB will not cause significant changes to citizenship fatigue and subsequent CWB. By contrast, employees with low POS lack of resources, they are more likely to suffer from resource loss and are more motivated to conserve resources (Kurtessis et al., 2017; Hobfoll et al., 2018). Therefore, when they engage in OCB at low levels to moderate levels, they mainly conserve their resources and induce citizenship fatigue. Then they will enter a defensive mode to preserve the self in which they are often aggressive and may become irrational, such as engaging in more CWB (Sommovigo et al., 2020). However, when they engage in OCB at moderate to high levels, the resource-acquisition tenet drives them to obtain new resources to stop the resource loss spiral (Hobfoll et al., 2018), As discussed above, they may decrease citizenship fatigue and subsequent CWB. Thus, employees with low POS will experience a stronger indirect effect of OCB on CWB through the mediation of citizenship fatigue than employees with high POS. Therefore, we propose the following moderated mediating hypothesis:

*Hypothesis 5. POS moderates the mediating effect of citizenship fatigue in the inverted U-shaped curvilinear relationship between OCB and CWB, and the mediating effect is stronger under conditions of low POS than high POS.*

## Materials and Methods

### Participants and Procedure

We aimed to recruit a heterogeneous sample from different industries for the present study to ensure sufficient variability in participants’ OCB, citizenship fatigue, POS and CWB (Faber and Walter, 2017). A three-waved longitudinal questionnaire survey with 1-month time intervals was conducted with full-time supervisors and subordinates. They were from different enterprises operating in China, involving industries of finance, consulting, IT and manufacture. We explained the purpose of the survey to the HR managers in each enterprise in advance. Then we asked them to determine the list of subordinates and supervisors who would like to participate in our study (for similar procedures, see Zhu et al., 2020). On request of the researcher, HR managers of each organization have a personal conservation with all the participants. The HR managers explained the purpose of the study, the discretionary nature of participation and the confidential treatment of the data. All these respondents indicated their willingness to participate by signing an informed consent. No incentives were provided for participation in the research. HR managers sent the questionnaires to most of the leaders and employees and got them back within sealed in envelopes. Besides, HR managers sent emails to a small number of participants who were not convenient to fill out paper questionnaires. Each respondent received a personal numeric code which allowed us to match employees’ questionnaires with supervisors’ ratings afterward.

To avoid the common method biases (Bauer et al., 1998), we collected data from employee-supervisor dyads in each branch in three waves. At Time 1, employees were asked to rate OCB, POS and demographic variables. After 1 month (at time 2), employees were asked to evaluate their own citizenship fatigue. After another month (at time 3), the corresponding supervisors were asked to report the followers’ CWB. We executed all the surveys during working hours. In total, we distributed surveys to 555 employees and 115 supervising managers. We excluded the questionnaires that could not be matched during three times and the following two types of invalid samples (the uncompleted questionnaires in which more than half of the single variable’s items were not answered, the questionnaires suspected to be answered carelessly). Finally, the sample consisted of 426 employees and 110 managers. The effective response rates were 76.8% for employees and 95.7% for supervisors.

For the employee sample, 44.8% were male, 75.1% had received a college degree or above, and 54.5% were married. The mean age and average work experience were 30.07 years old (*SD* = 4.54) and 3.62 (*SD* = 3.53) years, respectively. For the manager sample, 57.3% were male, 86.4% had received a college degree or above and 83.6% were married. The mean age and average work experience were 34.08 years old and 5.97 years, respectively.

### Measures

We conducted the study in China. We adopt the CWB scale directly since it was the Chinese language scale developed by Gao and Sun (2009). The Cronbach alpha of the scale was 0.9 (Gao and Sun, 2009). For the other English language scales (OCB scale, POS scale, citizenship fatigue scale), we translated the original English items to Chinese, strictly following a translation and back-translation procedure (Brislin, 1980). We adopted this procedure to maximize the equivalence between the translated scale and the original scale in terms of content and meaning. Specifically, Chinese versions were firstly translated by a bi-lingual (Chinese–English) translator who was familiar with the Chinese culture and the research topic. Afterward, the Chinese versions were back translated by another bi-lingual (Chinese–English) translator. And no major discrepancies were detected in the back translation. Finally, wording for certain measures was adjusted in minor ways to enhance the readability in China. We also checked for cultural sensitivities, to avoid cultural inappropriate translations and the similarity in meaning between the original and translated items. Before administrating the translated items, we tested the wording and meaning of the items with five employed graduate students who were not familiar with the research topic. In this manner, we ensured that participants could clearly understand all items. Indeed, the English scales have been applied in the Chinese context and reported good validity and reliability, including the OCB scale (e.g., Yam et al., 2017), citizenship fatigue scale (e.g., Liu and Yu, 2019), POS scale (e.g., Huang et al., 2021). All the variables were measured with seven-point Likert-type scales with the anchors “strongly agree” to “strongly disagree,” unless indicated otherwise. Internal consistency reliability coefficients were calculated for each scale and were provided along the diagonal in Table 1.

**TABLE 1:** Means, standard deviations and correlations.

Variables	Mean	*SD*	1	2	3	4	5	6	7	8	9
(1) Education	3.79	0.68									
(2) Gender	1.45	0.50	0.06								
(3) Age	30.07	4.54	–0.09	0.04							
(4) Marriage	1.54	0.50	−0.11*	0.03	0.56***						
(5) Work experience	3.62	3.53	−0.36***	–0.02	0.53***	0.36***					
(6) OCB	5.24	0.80	–0.09	−0.11*	0.16**	0.11*	0.20***	(0.90)			
(7) POS	4.80	0.86	0.04	−0.11*	0.08	0.08	0.12*	0.47***	(0.84)		
(8) Citizenship fatigue	2.94	1.09	0.02	0.01	–0.07	−0.12*	–0.03	0.02	−0.21***	(0.92)	
(9) CWB	1.84	0.78	0.04	0.16**	−0.10*	−0.18***	−0.21***	−0.14**	−0.15**	0.20***	(0.91)

N = 426. *p < 0.05; **p < 0.01; ***p < 0.001. Values in parentheses are reliabilities. POS, perceived organizational support. For education,1, “middle school or below;” 2, “high school;” 3, “college degree;” 4, “bachelor degree;” 5, “postgraduate degree and above”; For gender, 1, “male;” 2, “female”; For marriage, 1, “unmarried;” 2, “married.”

#### OCB

OCB was measured by a 16-item scale developed by Lee and Allen (2002). Sample items are “Keep up with developments in the organization” and “Help others who have been absent.” Items used in the present study include two facets of OCB, OCBO, and OCBI (Williams and Anderson, 1991; Geiger et al., 2019). OCBO includes behaviors that directly benefit the organization in general (e.g., puts in extra effort to aid the company, works extra hours), whereas OCBI refers to behaviors that immediately benefit specific individuals (e.g., helps others who have been absent; Williams and Anderson, 1991). OCB in this study was self-assessed by employees.

#### POS

Employees rated their POS using six items from Eisenberger et al. (2001). A sample item is “The company really cares about my living conditions.”

#### Citizenship Fatigue

Citizenship fatigue was measured by a six-item scale developed by Bolino et al. (2015). A sample item is “Because of going the extra mile for my organization, I feel on edge about various things.” Citizenship fatigue in our study was self-reported by employees.

#### CWB

CWB was measured by two dimensions, counterproductive work behavior-organizational (CWBO) and counterproductive work behavior-interpersonal (CWBI) (Robinson and Bennett, 1995). CWBI is a misbehavior that directly harms others (e.g., abuse), while CWBO is a misdemeanor directed toward the organization (e.g., slacking; Robinson and Bennett, 1995). Supervisors evaluated their subordinates’ CWB using the 9-item scale from Gao and Sun (2009). A sample item is “She/He is rude to her/his colleague.”

#### Control Variables

At the individual level, we controlled the demographic characteristics including employee’s gender, age, marriage, education and work experience because these variables often correlate with OCB or CWB. For example, some meta-analytic studies have showed that older employees are less likely to engage in CWB and more likely to engage in OCB (Ng et al., 2016; Pletzer, 2021), females engage in more OCB and less CWB than males (Ng et al., 2016; Thompson et al., 2020), work experience generally has negative correlations with CWB (Berry et al., 2007) and positive correlations with OCB (Wright and Bonett, 2002).

## Results

### Preliminary Analyses

#### Confirmatory Factor Analysis

Before testing our hypotheses, we conducted a series of confirmatory factor analyses (CFAs) using Mplus 7.4 (Muthén and Muthén, 2012) to examine the measurement model specifying OCB, POS, citizenship fatigue and CWB as four separate factors. Results suggested that the four-factor measurement model yielded a better model fit [Comparative Fit Index (CFI) = 0.91, Tucker-Lewis index (TLI) = 0.90, Standardized Root Mean Square Residual (SRMR) = 0.078, Root Mean Square Error of Approximation (RMSEA) = 0.06] than the three-factor model combined POS and OCB into one factor (CFI = 0.85, TLI = 0.83, SRMR = 0.09, RMSEA = 0.08), with a significant chi-square difference [Δχ^2^(3) = 615.52]; or the two-factor model with POS and OCB as one factor, and citizenship and CWB combined as the other factor [CFI = 0.75, TLI = 0.73, SRMR = 0.14, RMSEA = 0.10; Δχ^2^(5) = 1583.92]; or finally, the single-factor model [CFI = 0.60, TLI = 0.56, SRMR = 0.17; RMSEA = 0.12; Δχ^2^(6) = 3076.01].

According to Podsakoff et al. (2003), we used Harman’s single-factor test loading all of the four focal variables (OCB, POS, citizenship fatigue, CWB) in our study into an exploratory factor analysis (EFA) to test the common method bias (CMB). The result showed that the first factor of the unrotated principal component accounted for 23.5% of the variance in the four variables. After that, we further controlled the effects of method variance, adding method variance factors to the four-factor measurement model [CFI = 0.93, TLI = 0.92, SRMR = 0.06, RMSEA = 0.05; Δχ^2^(37) = 275.45]. According to Little (1997) and Cheung and Rensvold (2002), when the sample size was greater than 200, chi-square was too sensitive with changes of sample size and we should pay attention to the change of TLI. When the change of TLI was less than 0.05, adding method variance factors could not significantly improve the model fit. Our result showed that adding method variance factors to the four-factor measurement model only resulted in the increase of TLI by 0.02. These results indicated that there was no serious common method bias in this study.

#### Descriptive Statistics

Table 1 provided the descriptive statistics, correlations and reliability coefficients of the focal and control variables used in the study. Based on the Cronbach’s α coefficients, all scales exhibited internal consistency and ranged from 0.92 for citizenship fatigue to 0.84 for POS. Each latent variable scale exhibited acceptable internal consistency reliability (α > 0.70). We also analyzed the kurtosis and skewness values for the OCB, POS, citizenship fatigue and CWB. The kurtosis values for OCB, POS, citizenship fatigue and CWB were −0.350, 0.244, 0.277, and 0.778, respectively. The skewness values for OCB, POS, citizenship fatigue and CWB were −0.292, −0.246, −0.590, and 0.059, respectively. Gender was negatively related to OCB (*r* = −0.11, *p* < 0.05) and positively related to CWB (*r* = 0.16, *p* < 0.01). Age was positively related to OCB (*r* = 0.16, *p* < 0.01), and negatively related to CWB (*r* = −0.10, *p* < 0.5). Marriage displayed a positive correlation with OCB (*r* = 0.11, *p* < 0.05), was negatively related to citizenship fatigue (*r* = −0.12, *p* < 0.05) and negatively related to CWB (*r* = −0.18, *p* < 0.001). Work experience was positively related to OCB (*r* = 0.20, *p* < 0.001) and negatively related to CWB (*r* = −0.21, *p* < 0.001). The linear relationship between OCB and citizenship fatigue was not significant (*r* = 0.02, *ns*). OCB was negatively correlated with CWB (*r* = −0.14, *p* < 0.01), and citizenship fatigue was significantly positively correlated with CWB (*r* = 0.20, *p* < 0.001), indicating the need for non-linear analysis.

### Hypotheses Testing

We used hierarchical regression analysis (Baron and Kenny, 1986) and polynomial regression analyses (Edwards and Lambert, 2007) in SPSS 25.0 and MPLUS 7.4 to test our hypotheses. The results of analyses predicting citizenship fatigue and CWB are reported in Table 2. We grand-mean-centered the independent variable (OCB) and moderator (POS) to reduce unnecessary multicollinearity between the linear terms and their quadratic counterparts before evaluating the regression equations (Cohen et al., 2003). Followed Cohen et al.’s (2003) procedure and Dawson’s (2014) method and consistent with common practice in examining moderating effects in the context of curvilinear relationship (e.g., Faber and Walter, 2017; Li et al., 2019; Montani et al., 2020), predictors were entered into the regression equation for citizenship fatigue in the following order: (a) control variables, (b) OCB, (c) the quadratic term of OCB squared, (d) the linear interaction between OCB and POS, the interaction between OCB squared and POS.

**TABLE 2:** Results of hierarchical regression analyses.

	CF	CF	CF	CF	CWB	CWB	CWB	CWB
Variables	Model 1	Model 2	Model 3	Model 4	Model 5	Model 6	Model 7	Model 8
Education	0.01	0.01	0.05	0.07	–0.06	–0.06	–0.05	–0.05
Gender	0.02	0.02	–0.00	–0.00	0.16**	0.15**	0.14**	0.14**
Age	–0.02	–0.02	–0.03	–0.05	0.08	0.09	0.09	0.10
Marriage	–0.11	–0.11	–0.10	–0.09	−0.16**	−0.16**	−0.15**	−0.13*
Work experience	0.02	0.02	0.05	0.06	−0.22***	−0.21**	−0.19**	−0.20**
OCB		0.03	–0.05	–0.02		–0.09	−0.13*	−0.12*
OCB^2^			−0.29***	−0.26***			−0.13**	–0.09
POS				−0.35***				
OCB × POS				–0.00				
OCB^2^ × POS				0.24**				
CF								0.16**
*R* ^2^	0.01	0.01	0.09	0.15	0.09	0.09	0.11	0.13
Δ*R*^2^		0.00	0.08***	0.06***		0.00	0.02**	0.02**
ΔF		0.47	34.80***	9.92***		3.42	7.14**	10.68**

N = 426. CF, citizenship fatigue; POS, perceived organizational support. *p < 0.05; **p < 0.01; ***p < 0.001.

Hypothesis 1 predicted OCB had an inverted U-shaped relationship with citizenship fatigue. As shown in Model 2 of Table 2, OCB was not significantly related to citizenship fatigue (β = 0.03, *ns*). In Model 3, the quadratic terms of OCB squared was significantly negative related to citizenship fatigue (β = −0.29, *p* < 0.001, Δ*R*^2^ = 0.08). To further test Hypothesis 1, using MPLUS software, based on 5000 bootstrap samples, we found that the coefficient of quadratic terms of OCB squared on citizenship fatigue was −0.383 [95 percent confidence interval (CI) = [−0.496, −0.254]]. Because the CI excluded zero, Hypothesis 1 was supported.

**FIGURE 2:**
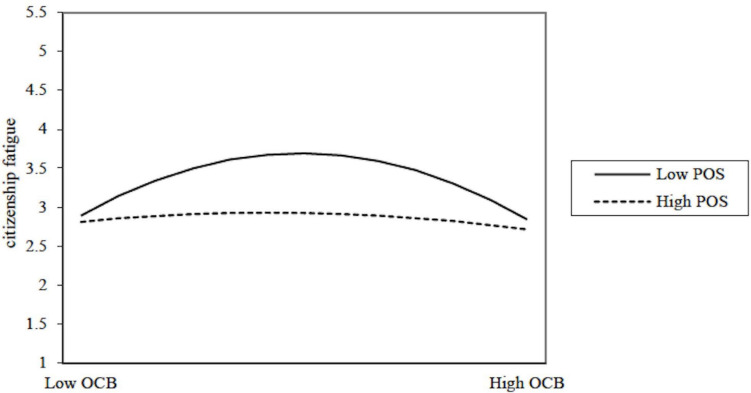
The moderating role of POS on the relationship between OCB and citizenship fatigue.

Moreover, as shown in Model 4 of Table 2, the interaction term between quadratic OCB squared and POS was significant (β = 0.24, *p* < 0.01, Δ*R*^2^ = 0.06). Based on 5000 bootstrap samples, we found that the coefficient of the interaction between quadratic OCB squared and POS on citizenship fatigue was 0.27 (95% CI = [0.098, 0.450]). Because the CI excluded zero, Hypothesis 2 was supported, indicating that POS moderated the inverted U-shaped relationship between OCB and citizenship fatigue. To test Hypothesis 2 more accurately, we performed the simple slopes of the regression lines corresponding to the possible combinations of different levels of OCB with high (mean+1SD) and low levels of POS (mean−1SD) (Aiken and West, 1991). Results of simple slope tests showed that, in the case of high POS, the simple slope of the regression lines was not significant for citizenship fatigue at low level of OCB (γ = 0.14, *ns*) and high level of OCB (γ = −0.22, *ns*). By contrast, in the case of low POS, the low level of OCB was positively related to citizenship fatigue (γ = 0.90, *p* < 0.001), the high level of OCB was negatively related to citizenship fatigue (γ = −0.94, *p* < 0.01). To facilitate the interpretation of this quadratic-by-linear interaction effect, we visualized the interaction in Figure 2. As shown in the Figure 2, when POS was high, OCB was not related to citizenship fatigue. When POS was low, there was an inverted U-shaped relationship between OCB and citizenship fatigue. Thus, Hypothesis 2 was supported.

Table 2 also shows the results of regression analysis for CWB. Consistent with common practice in examining mediating effects in the curvilinear relationship (e.g., Montani et al., 2020), predictors were entered into the regression equation for CWB in the following order: (a) control variables, (b) OCB, (c) the quadratic term of OCB squared, (d) citizenship fatigue. As shown in Model 8 of Table 2, using gender, age, marriage, education and work experience as covariates, OCB, the quadratic term of OCB squared, and citizenship fatigue as other predictors, we found that citizenship fatigue was positive correlated to CWB (β = 0.16, *p* < 0.01), providing support for Hypothesis 3. Our model also involved testing the non-linear mediation effects from OCB to CWB with citizenship fatigue as a mediator (Hypothesis 4). Model 7 showed that OCB had an inverted U-shaped relationship with CWB as the coefficient for OCB squared was significant (β = −0.13, *p* < 0.01, Δ*R*^2^ = 0.02). Based on 5000 bootstrap samples, we found that the coefficient of OCB squared on CWB was −0.122 (95% CI = [−0.199, −0.048]), the CI excluded zero. As shown in Model 8 of Table 2, when citizenship fatigue is entered, OCB had an inverted relationship with CWB (β = −0.09, ns, Δ*R*^2^ = 0.02). This indicated that citizenship fatigue fully mediated the inverted U-shaped curvilinear relationships between OCB and CWB, supporting H4.

Additionally, to calculate the curvilinear indirect effect for Hypothesis 4, we followed the procedures introduced by Hayes and Preacher (2010) and modified by Li et al. (2019) and Montani et al. (2020). Specifically, we state the procedures for testing curvilinear indirect effects in detail as follows. Hayes and Preacher (2010) explained that a curvilinear mediation effect is a particular case of an indirect effect in which an independent variable is non-linearly associated with a mediator, and in turn, linearly related to a dependent variable. They used θ to denote the rate at which a change in the independent variable changes the dependent variable indirectly through changes in the mediator (Hayes and Preacher, 2010). To calculate the instantaneous indirect effect θ, they calculated the first partial derivation of the function of the mediator with respect to the independent variable (Hayes and Preacher, 2010). Then, they calculated the first partial derivation of the function of the dependent variable with respect to the mediator (Hayes and Preacher, 2010). θ can be estimated as the product of the two constructs. For this study, the formula can be described as:

(1) $$\begin{array}{c} \theta =\frac{\partial \left(citizenshipfatigue\right)}{\partial \left(OCB\right)}\frac{\partial \left(CWB\right)}{\partial \left(citizenshipfatigue\right)} \end{array}$$

Based on Hypothesis 1, we used the Equation (2) to depict the relationship between OCB and citizenship fatigue:

(2) $$\begin{array}{c} \mathrm{Citizenship}\mathrm{fatigue}=\beta_{0}+\beta_{1}\left(OCB\right)+\beta_{2}\left(OCBsquared\right)+\sigma \end{array}$$

According to Hypothesis 3 and Hypothesis 4, CWB is linearly related to citizenship fatigue and curvilinear related to OCB:

(3) $$\begin{array}{c} \text{CWB}=\alpha_{0}+\alpha_{1}\left(\text{OCB}\right)+\alpha_{2}\left(OCBsquraed\right)+\alpha_{3}\left(citizenshipfatigue\right)+\sigma \end{array}$$

Therefore, when we test the mediation effect in Hypothesis 4, we can derive the partial derivative of citizenship fatigue with respect to OCB from Equation (2), and likewise, derive the partial derivative of CWB with respect to citizenship fatigue from Equation (3). According to Equations (1), (2), and (3), the instantaneous indirect effect of OCB on CWB through citizenship fatigue is:

(4) $$\begin{array}{c} \theta_{1}=\left[\beta_{1}+2\beta_{2}\left(OCB\right)\right]\times \alpha_{3} \end{array}$$

As shown in Equation (4), θ_1_ is a linear function of OCB. If θ_1_ at low and high levels of OCB (i.e., one standard deviation below the mean, one standard deviation above the mean) is significant, the Hypothesis 4 of curvilinear mediation effect will be supported. We used SPSS MEDCURVE macro (Hayes and Preacher, 2010) to calculate the bias-corrected bootstrap 95% confidence intervals for the curvilinear indirect relationships between OCB and CWB via citizenship fatigue. As shown in Table 3, based on 5000 bootstrap samples, when the level of OCB was low, the indirect effect was 0.061 (95% CI = [0.022, 0.107]). Conversely, when the level of OCB was high, the indirect effect was −0.075 (95% CI = [−0.147, −0.024]). Because the CI excluded zero, Hypothesis 4 was supported, indicating that citizenship fatigue had a mediation effect between OCB and CWB.

**TABLE 3:** The mediating effect of citizenship fatigue on the relationship between OCB and CWB.

Mediator	OCB	SE	Indirect effect (θ)	95% BC confidence LL	95% BC confidence UL
Citizenship fatigue	Low	0.022	0.061	0.022	0.107
	high	0.032	−0.075	−0.147	−0.024

*N = 426. Low = 1 SD below the mean, High = 1 SD above the mean. Bootstrapping sample size = 5000. SE, standard error; LL, bootstrapping lower limit confidence interval; UL, bootstrapping upper limit confidence interval.*

Hypothesis 5 involved the curvilinear moderated mediation effects of POS on CWB. Edwards and Lambert (2007) suggested establishing polynomial regression equations and models for testing moderated mediation. Maruping et al. (2015) combined Edwards and Lambert’s (2007) and Hayes and Preacher’s (2010) procedures to test a moderated-mediation model. Following their works, to analyze Hypothesis 5, we conducted a moderated analysis by a test of instantaneous indirect effects at varying levels of POS. Specifically, we took the moderation role of POS into analysis. According to Hypothesis 2, we used the Equation (5) to depict the moderating effect of POS:

(5) $$\begin{array}{c} \mathrm{Citizenship}\mathrm{fatigue}=\beta_{3}+\beta_{4}\left(OCB\right)+\beta_{5}\left(OCBsquared\right)+\beta_{6}\left(POS\right)+\beta_{7}\left(OCB\times POS\right)+\beta_{8}\left(OCBsquared\times POS\right)+\sigma \end{array}$$

According to Equations (1), (3), and (5), we estimated instantaneous indirect effects for OCB on CWB (through citizenship fatigue) under weak and strong POS:

(6) $$\begin{array}{c} \theta_{2}=[\beta_{4}+2\beta_{5}\left(OCB\right)+\beta_{7}\left(POS\right)+2\beta_{8}\left(OCB\times POS\right)]\times \alpha {}_{3} \end{array}$$

As shown in Equation (6), θ_2_ is not constant, but a linear function of OCB, POS and the product term (*OCB*×*POS*). Mathematically, if the difference in θ at low (one standard deviation below the mean) versus high (one standard deviation above the mean) levels of OCB and POS is significantly different from zero, the Hypothesis 5 of moderated mediation effect will be supported. Specifically, we used Mplus software (Muthén and Muthén, 2012) to calculate the bootstrap 95% confidence intervals for the instantaneous indirect effects between OCB and CWB via citizenship fatigue at high and low levels of POS. As shown in Table 4, the differences in θ for low OCB when POS was low versus high was 0.084 (95% CI = [0.017, 0.204]). Similarly, the differences in θ for high OCB when POS was low versus high was −0.080 (95% CI = [−0.200, −0.017]). The CI for the two differences scores was 0.164, which excluded zero (95% CI = [0.045, 0.378]). It meant that the difference in θ at high and low levels of OCB for employees with high levels of POS was significantly different for those with low levels of POS. Therefore, the results showed that POS moderates the mediating effect of citizenship fatigue in the inverted U-shaped curvilinear relationship between OCB and CWB, and the mediating effect is stronger under conditions of low POS than high POS. Hypothesis 5 was supported.

**TABLE 4:** The conditional indirect effect of OCB on CWB through citizenship fatigue.

Moderator	Predictor		CWB		
POS	OCB	SE	Indirect effect (θ)	95% BC confidence LL	95% BC confidence UL
Low	Low	0.036	0.100	0.036	0.182
Low	High	0.050	−0.104	−0.231	−0.028
High	Low	0.027	0.015	−0.032	0.078
High	High	0.022	−0.025	−0.082	0.010
Difference	Low	0.046	0.084	0.017	0.204
Difference	High	0.044	−0.080	−0.200	−0.017

*N = 426. POS, perceived organizational support; Low, 1 SD below the mean; High, 1 SD above the mean; Bootstrapping sample size = 5000. SE, standard error; LL, bootstrapping lower limit confidence interval; UL, bootstrapping upper limit confidence interval.*

## Discussion

Drawing on COR theory, we developed and tested a moderated mediation model to examine whether, how, and when OCB has a curvilinear effect on citizenship fatigue and influences CWB. Specifically, empirical results from a multi-wave and multi-source study showed that OCB had an inverted U-shaped curvilinear relationship with CWB, and citizenship fatigue mediated the direct relationship. Furthermore, POS moderated the relationship between OCB and citizenship fatigue, such that the relationship was insignificant when POS was high and was inverted U-shaped when POS was low. POS moderates the mediating effect of citizenship fatigue in the inverted U-shaped curvilinear relationship between OCB and CWB. These findings have important theoretical and practical implications, which are discussed below.

### Theoretical Contributions

Our research has the following theoretical contributions. First, our research took a step further toward resolving the lack of consistent findings about the relationship between OCB and CWB yielded by prior studies. Most previous studies focused on a linear relationship (e.g., Dalal, 2005), and inconsistent results might stem from that approach. Our findings suggested that the paradoxical results could be explained not only by identifying its boundary conditions (i.e., external motivation; Yam et al., 2017), but also by modeling a curvilinear effect of OCB on CWB. The research found an inverted U-shaped relationship between OCB and CWB through citizenship fatigue. Specifically, when people engage in OCB at low to moderate levels, they conserve resources and are unable to obtain new resources, which induces an increase in citizenship fatigue and subsequent CWB (Hobfoll et al., 2018). By contrast, when people engage in OCB at moderate to high levels, the resource acquisition motivation drives them to obtain resources, which decreases citizenship fatigue and restrains CWB (Hobfoll et al., 2018). From a theoretical perspective, this suggests the resource conservation tenet and resource acquisition motivation may depend on the level of OCB.

Second, we answer recent calls to examine the role of resource depletion in the relationship between OCB and CWB (Bolino and Klotz, 2015). Scholars have suggested that OCB is a resource-depleting activity and that when employees’ self-control resources are depleted, they are especially likely to engage in CWB (Koopman et al., 2020). Indeed, some studies provided evidence to that regard in helping behavior research. For example, one study found that helping behavior leads to workplace deviance through resource depletion (Xiao et al., 2021). However, these studies adopted a linear explanation. Drawing on COR theory, our study demonstrated that citizenship fatigue—described as a state when people’s resources are overstretched or exhausted—plays a mediating role in the curvilinear effect of OCB on CWB. Therefore, apart from the moral licensing theory (e.g., Yam et al., 2017) and negative emotion perspective (Spector and Fox, 2010a,b), we found that COR theory was another important mechanism linking OCB and CWB, which was notably absent in the literature on OCB and CWB. Thus, this research broadens the underlying theory for explaining how and when good soldiers might behave unethically in a non-linear way.

Third, drawing from COR theory (Hobfoll, 1989, 2001; Hobfoll et al., 2018), we extended the research on citizenship fatigue by examining the curvilinear relationship between OCB and citizenship fatigue. Our study found that there was an inverted U-shaped relationship between OCB and citizenship fatigue. This finding expanded the previous explanation of citizenship fatigue and even other states of resource loss (e.g., emotional exhaustion) addressed by COR theory (Halbesleben, 2010). Past studies on citizenship fatigue have mainly discussed the dark side of OCB according to the resource-depleting feature (e.g., Qiu et al., 2020), arguing that employees felt tired when they engaged in OCB because of the continuous consumption of resources (Bolino et al., 2015; Liu and Yu, 2019). However, our study took the resource investment mechanisms of COR theory into consideration. The results have shown that employees will engage in OCB to gain resources when they feel exhausted (Halbesleben and Bowler, 2007). Additionally, according to the gain paradox principle and resource loss spiral corollary of COR theory, employees are eager to gain new resources to stop further resource loss spiral and prevent them from health impairment in the context of resource loss (Hobfoll et al., 2018). Indeed, the curvilinear relationship between OCB and citizenship fatigue also indicates that the distinction between the resource conservation tenet and resource investment tenet may depend on the level of OCB. Our study showed that when employees engaged in a high level of OCB, the employees’ resource acquisition tenet was triggered, where they would gain enough resources, and led to a decrease in the degree of citizenship fatigue. Furthermore, although previous studies revealed the costs and benefits of OCB on individual well-being (e.g., job satisfaction, affective commitment), they reported the effects in a linear rather a curvilinear manner and investigated the mechanisms independently (Koopman et al., 2016; Koopman et al., 2020). As such, our study also offers new sights to clarify the extent to which OCB exerts a motivating and energizing, rather than health-impairing, effect on various work outcomes.

Finally, this study deepens our knowledge of the impact of organizational resources such as POS on the relationship between OCB and citizenship fatigue. COR theory posits that individuals with greater resources are less susceptible to resource loss and more capable of acquiring new resources given their tendency to accumulate resources (Hobfoll et al., 2018). Our study showed that POS was a key resource that fosters the beneficial effect of the resource acquisition mechanism of OCB and buffers the impairing effect of the resource-depleting mechanism of OCB. Although a prior study has investigated the moderating role of POS between OCB and citizenship fatigue (Bolino et al., 2015), the research only considered the resource-depleting mechanism of OCB and assumed a linear relationship between OCB and citizenship fatigue. Our study found that POS moderated the relationship between OCB and citizenship fatigue, such that the relationship was insignificant when POS was high and inverted U-shaped when POS was low. This finding extends our understanding of the impact of POS in the resource-depleting and resource-acquiring processes.

### Practical Implications

Our results also provide several practical implications for organizations. First, moderate levels of OCB will induce the highest level of CWB according to the inverted U-shaped relationship between OCB and CWB. Thus, encouraging individuals to engage in OCB may not always lead to positive outcomes (Koopman et al., 2016). A moderate level of OCB might intensify employees’ perception of resource depletion and motivate them to engage in CWB to counteract stress. This finding suggests that managers should pay attention to controlling the level of OCB (e.g., enact measures to help employees deal with potential resource loss). HR managers should be aware that excessive expectations can have negative repercussions for employees’ emotional state (Koopman et al., 2020). Therefore, HR professionals must not force employees into such volunteerism (Koopman et al., 2020). One potential measure could be designing monitoring systems to avoid subordinates’ citizenship fatigue (Klotz et al., 2018).

Second, organizations should recognize that employees can take action to obtain new resources to reduce citizenship fatigue. Once employees obtain new resources, they may recover from resource loss (Hobfoll et al., 2018). Therefore, managers should support employees in dealing with citizenship fatigue by helping them gain new resources, such as by praising employees (Grobelna, 2021). Employees might be reluctant to admit that they feel stressed when their supervisors encourage them to engage in OCBs, to avoid being perceived as weak or ungrateful for their current employment (Bergeron, 2007). HR managers should be proactive in monitoring whether their expectations that employees take on additional responsibilities have become excessive (Koopman et al., 2020). Specifically, they should create a culture in which employees feel comfortable expressing their concerns about work pressures as well as develop procedures to enable employees to share their workloads (De Clercq et al., 2019). For example, they should create guidelines for how employees can support one another in achieving the combined execution of their extra-role activities, depending on their respective skill sets and capabilities (De Clercq et al., 2019). For example, targeted initiatives could encourage experienced employees to support newcomers’ efforts to cope with citizenship fatigue through one-on-one mentoring (Grobelna, 2021). Moreover, periodic interventions such as job demand-resource intervention, could be conducted to help employees build and maintain their personal and job resources and subsequently deal with citizenship fatigue (Liu and Yu, 2019).

Finally, our findings suggested that employees with high POS do not feel pressure and do not engage in CWB. When engaging in OCB, employees with high POS may have abundant resources to cope with stress. Therefore, organizations should create a comfortable and supportive organizational environment for employees to increase their perceptions of organizational support (Garcia et al., 2021). On the one hand, organizations should adopt targeted procedures and human resources practices (Caesens et al., 2019). Prior studies have indicated that perceptions of organizational support can be fostered by rendering fair decisional policies, maintaining open channels of communication with employees (Eisenberger and Stinglhamber, 2011), assuring employees that their jobs are secure, offering valuable training or developmental programs that promote employees’ personal growth, and eliminating continual work overloads (Rhoades and Eisenberger, 2002). Thus, organizations should care about employees’ well-being, value their contributions, and be willing to help employees when they need a favor (She et al., 2019). On the other hand, managers should support their subordinates, for instance, by having regular meetings with them, resolving any conflicting job responsibilities, or providing them with the materials or emotional resources they need (e.g., Eisenberger and Stinglhamber, 2011). Managers should create an internal environment in which employees feel motivated to perform OCB, by communicating that OCB provides valuable and unique opportunities for personal development and growth (De Clercq et al., 2019).

### Limitations and Future Directions

Despite our contributions, our study has several limitations. First, OCB was rated by employees, which may lead to some biases. In the future, employees’ supervisors can evaluate the frequency of OCB to further test our model.

Second, our study only measured one type of OCB. As stated earlier, items used in the present study included OCB directed at the organization and OCB directed at individuals (Williams and Anderson, 1991). However, there are other categories and types of OCB, such as sportsmanship (Organ, 2018). Therefore, future research should also examine the impact of other types of OCB on citizenship fatigue.

Third, although our study combined the resource conservation tenet and resource acquisition tenet to emphasize the relationship between OCB and citizenship fatigue, we did not measure actual resource loss resulting from OCB. Another limitation is that we did not control for the existing moral mechanism based on the perspective of moral licensing theory (e.g., Yam et al., 2017). To highlight the unique role of the resource depletion perspective, future research should test the mediating role of citizenship fatigue under the premise of controlling for this moral mechanism and resource loss.

In addition, an empirical limitation of this study pertains to the consideration of only five control variables (gender, age, marriage, education, and work experience) in the statistical models. Indeed, some studies have argued that type of industries may influence the findings because service industry may have some special requirements on OCB (Ocampo et al., 2018; Qiu et al., 2019). Future research could consider whether our results hold even when controlling for other factors that might determine employees’ responses to the hardships of OCB and resource-allocation, such as their task performance (Bergeron, 2007; Bolino et al., 2015), part-time status (Deery et al., 2017), and industry type (Ocampo et al., 2018; Qiu et al., 2019).

Moreover, in terms of the boundary conditions of OCB on citizenship fatigue, we only tested the moderating role of POS, but other factors may influence the relationship between OCB and citizenship fatigue, including personal characteristics such as emotional stability (Liu and Yu, 2019), mindfulness (Montani et al., 2020), and moral identity (Aquino, 2002; Skarlicki et al., 2008), as well as different social support forms such as supervisor support (Sommovigo et al., 2019) and organizational climate (Organ, 2018). Therefore, future research should incorporate these moderators into the research framework to gain a deeper understanding of the effects of OCB on CWB.

Finally, relying on data from one country, China, might limit the generalizability of the findings. The present results from the Chinese context may be not applicable to the western contexts. In fact, prior studies indicated that cross-cultural differences among Asiatic and American employees may result in different reactions (e.g., Shao and Skarlicki, 2014), and differences have been found in the underlying mechanisms explaining unethical behavior among geographically close nations (Sommovigo et al., 2020), suggesting the importance of considering omnibus contextual influences (Johns, 2006). Therefore, future research should conduct cross-cultural research to include western cultures.

## Conclusion

Unlike the traditional view of the linear relationship between OCB and CWB, our study integrated the resource conservation tenet and resource acquisition tenet of COR theory to explore the curvilinear relationship between OCB with CWB. It also examined the internal mechanism and boundary condition of the relationship between OCB and CWB.

As hypothesized, the results indicated that there was an inverted U-shaped relationship between OCB and citizenship fatigue. We also found that POS moderated the relationship between OCB and citizenship fatigue, such that the relationship was insignificant when POS was high and was inverted U-shaped when POS was low. Additionally, there was a positive linear relationship between citizenship fatigue and CWB. Citizenship fatigue mediated the relationship between OCB and CWB. POS moderates the mediating effect of citizenship fatigue in the inverted U-shaped curvilinear relationship between OCB and CWB, and the mediating effect is stronger under conditions of low POS than high POS.

## Data Availability Statement

The original contributions presented in the study are included in the article/supplementary material, further inquiries can be directed to the corresponding author/s.

## Ethics Statement

Institutions in China do not have Institutional Review Board. The protocol was approved by the Renmin University of China. In the survey process, all participants were informed that participation was voluntary and assured that their responses would be only used for our research and kept strictly confidential. The patients/participants provided their written informed consent to participate in this study.

## Author Contributions

FX and SX developed the theoretical model and hypotheses. FX collected the data and wrote the manuscript. JZhu, JZhou, BZ, and CY provided comments on different versions of the manuscript and edited the manuscript. All authors contributed to the article and approved the submitted version.

## Conflict of Interest

The authors declare that the research was conducted in the absence of any commercial or financial relationships that could be construed as a potential conflict of interest.

## Publisher’s Note

All claims expressed in this article are solely those of the authors and do not necessarily represent those of their affiliated organizations, or those of the publisher, the editors and the reviewers. Any product that may be evaluated in this article, or claim that may be made by its manufacturer, is not guaranteed or endorsed by the publisher.
